# One-Step Initial Alignment Algorithm for SINS in the ECI Frame Based on the Inertial Attitude Measurement of the CNS

**DOI:** 10.3390/s22145123

**Published:** 2022-07-08

**Authors:** Jun Tang, Hongwei Bian, Heng Ma, Rongying Wang

**Affiliations:** 1 The Department of Navigation Engineering, Naval University of Engineering, Wuhan 430033, China; tjdegree@163.com (J.T.); mkggrr2004@aliyun.com (H.M.); wry441@163.com (R.W.); 2 The Department of Navigation, Dalian Naval Academy, Dalian 116018, China

**Keywords:** initial alignment, misalignment angle, attitude measurement, SINS, CNS

## Abstract

To solve the problem of high-precision and fast initial alignment for the Strapdown Inertial Navigation System (SINS) under both dynamic and static conditions, the high-precision attitude measured by the celestial navigation system (CNS) is used as the reference information for the initial alignment. The alignment algorithm is derived in the Earth-centered inertial (ECI) frame. Compared with the alignment algorithm in the navigation frame, it is independent of position parameters and avoids the influence of the approximate error caused by the dynamic deflection angle. In addition, hull deformation is considered in attitude optimal estimation, which can realize initial the alignment of the SINS installed in various parts of the carrier. On this basis, the velocity measurement information is added to the alignment process, which further improves the accuracy and speed of the initial alignment under static conditions. The experimental results show that the algorithms proposed in this paper have better performance in alignment accuracy, speed, and stability. The attitude and velocity matching algorithm in the ECI frame can achieve alignment accuracy better than 0.6′. The attitude matching algorithm in the ECI frame has better robustness and can be used for both dynamic and static conditions, which can achieve alignment accuracy better than 1.3′.

## 1. Introduction

Initial alignment of the SINS is an indispensable step before the pure navigation calculation stage. The main purpose of the initial alignment is to determine the initial attitude [1]. In the military field, the performances of anti-interference, rapidity, and robustness in complex conditions for the initial alignment have become the focus of attention [2,3].

The past few decades have seen fruitful algorithms development for the SINS initial alignment. According to the reference information sources, these methods can be loosely grouped into two main categories: self-alignment and unself-alignment. The self-alignment without external information has drawn particular attention due to its military application value. At present, the traditional two-stage alignment method is commonly adopted for self-alignment. The method is composed of coarse alignment and fine alignment, which can only be accomplished under stationary or mooring conditions [4,5,6,7]. The severe noise inherent in the inertial measurement unit (IMU) outputs may degrade the alignment performance in the coarse alignment process. The fine alignment based on the linear or approximately linear error equations necessitates the misalignment angle, which is not so large after coarse alignment. Therefore, the urgent problem to be solved for two-stage alignment is to shorten the total alignment time and improve the accuracy of coarse alignment, which promotes the development of the nonlinear alignment method based on nonlinear error equations [8,9]. The key to this method is to establish the nonlinear dynamic model, and use nonlinear filtering methods to perform the alignment based on the established nonlinear model. In this respect, the method is not limited by the size of the misalignment angle, and the coarse alignment is no longer needed. Although the nonlinear initial alignment method has some advantages in alignment speed, it is difficult to reach the alignment precision by the traditional two-stage method. Chang conducted in-depth research on nonlinear initial alignment methods [10,11,12]. In his research, the dynamic model based on nonlinear error equations was re-derived with consideration of the mismatch between the calculated navigation frame and the actual navigation frame [13,14,15,16]. The unself-alignment requires external information as the reference for the initial alignment [17,18,19,20,21]. According to the current status of research, the external reference information is usually provided by GPS, log, or a high-precision main inertial navigation system. This method is an optimal estimation process based on external measurement information, so in theory, it has higher alignment accuracy and faster alignment speed and can be applied to both dynamic and static conditions [22]. However, GPS fails to provide high-precision attitude information directly, which will lead to low accuracy of the azimuth misalignment angle estimation, and the information provided by GPS is prone to interference [23]. The measurement accuracy of the log will be affected under high dynamic and deep water conditions, and the Doppler log needs to send pulse signals outward [24]. In the field of military applications, concealment is a problem that must be considered [25,26]. In addition, the reference information provided by the master inertial navigation system contains the inherent alignment error and accumulated error, which can also affect the alignment accuracy [27,28,29].

To summarize, the contradiction between the initial alignment accuracy and speed has not been completely solved, the reference information of the initial alignment is relatively single, which has certain defects in autonomy and anti-interference. The CNS—taking natural celestial bodies as observation targets—is an autonomous navigation system. It can directly provide high-accuracy attitude information in the ECI frame without the help of any external navigation information [30]. The significant advantages of such systems are the high-precision of attitude measurement, immunity to electromagnetic interference, and no navigation error accumulation over time, such as SINS. In theory, the attitude measurement accuracy of the CNS can reach several arc seconds, and the positioning accuracy can be better than 10 m [31,32]. With the rapid development of optical sensors, the applicability of the CNS has been greatly improved, so its application value in the military field is more prominent. With this consideration, this paper is devoted to investigating the one-step initial alignment based on the inertial attitude measurement of the CNS under both dynamic and static conditions.

The highlights of this paper are as follows.

1. The high-precision attitude measured by the CNS is used as the reference information for the initial alignment. Compared with the traditional initial alignment algorithm, the algorithm proposed in this paper has better performance in alignment accuracy, speed, and stability.
2. Compared with the alignment dynamic model in the navigation frame, the model established in the ECI frame is more simplified and avoids the influence of the approximate error caused by the dynamic deflection angle.
3. The attitude matching algorithm in the ECI frame proposed in this paper is independent of position parameters and can be used for both dynamic and static conditions.
4. Based on the attitude matching algorithm, the velocity measurement information is added to the optimal estimation process to form the attitude and velocity matching algorithm, which further improves the accuracy and speed of the initial alignment under static conditions.
5. The hull deformation is considered in the attitude optimal estimation, which can realize a fast and high-precision initial alignment of the SINS installed in various parts of the carrier.
6. The CNS is immune to electromagnetic interference and has no navigation error accumulation over time, such as SINS, so the algorithms based on the CNS have important military application values and wider applicability.

The paper is organized as follows. The frame definition and initial alignment system composition are introduced in Section 2. The attitude measured by the CNS serves as the reference information for the initial alignment; the attitude measurement principle is introduced in Section 3. In Section 4, we deduce the initial alignment model in the navigation frame, and the existing problems are analyzed. The attitude matching and attitude velocity matching algorithm in the ECI frame is established, respectively, in Section 5 and Section 6. Section 7 presents the experimental results. Section 8 summarizes the whole paper.

## 2. Frame Definition and Composition of the Initial Alignment System

The relevant frames are defined as follows.

The Earth-centered Earth-fixed (ECEF) frame is defined as e.

The Earth-centered inertial (ECI) frame is defined as i.

The calculated Earth-centered inertial frame is defined as $i^{\prime }$.

The misalignment angle from i to $i^{\prime }$ is defined as $\phi_{i,i^{\prime }}^{}$.

The navigation frame is defined as n.

The calculated navigation frame is defined as $n^{\prime }$.

The misalignment angle from n to $n^{\prime }$ is defined as $\phi_{n,n^{\prime }}^{}$.

The body frame in the position of the SINS is defined as bm.

The calculated body frame in the position of the SINS is defined as $bm^{\prime }$.

The misalignment angle from bm to $bm^{\prime }$ is defined as $\phi_{bm,bm^{\prime }}^{}$.

The body frame in the position of the CNS is defined as b.

The CNS frame is defined as s.

The installation error angle from s to b is defined as $\mu_{s,b}^{}$.

The frame formed by the static deformation angle $\mu_{b,ba}^{}$ deviated from b is defined as ba.

The dynamic deflection deformation angle deviated from ba to bm is defined as $\gamma_{ba,bm}^{}$.

**Figure 1:**
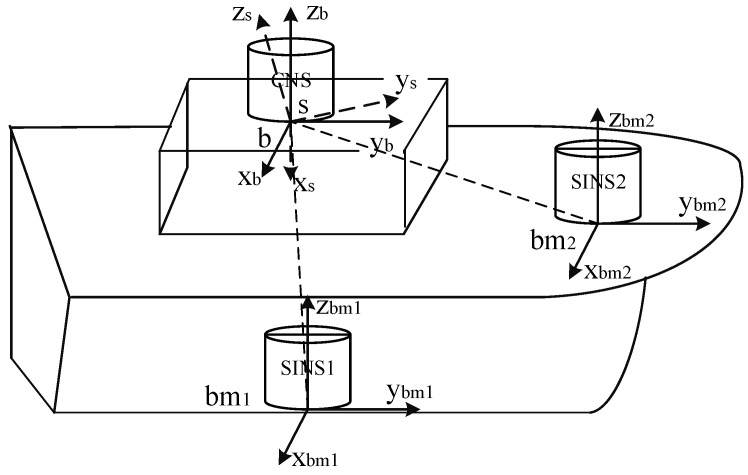
The composition of the initial alignment system.

The composition of the initial alignment system is shown in Figure 1. The CNS is usually installed on the top of the carrier platform, which is not consistent with the body frame of the SINS installation position. The deviation angle between b and $bm_{1}$ or $bm_{2}$ is composed of the static deformation angle $\mu_{b,ba}^{}$ and the dynamic deflection deformation angle $\gamma_{ba,bm}^{}$ [33]. The transformation from s to b should also consider the influence of the installation error angle $\mu_{s,b}^{}$ of the CNS. 

## 3. Attitude Measurement Principle of the CNS

${\tilde{R}}_{j}^{s}=[x_{j}\;y_{j}\;z_{j}{]}^{T}(\;j=1,2,\cdots ,m)$ are the coordinates of stars in frame s, which are obtained by observing m stars with the star sensor. At the same time, ${\tilde{R}}_{j}^{i}=[X_{j}\;Y_{j}\;Z_{j}{]}^{T}(\;j=1,2,\cdots ,m)$, the coordinates of stars in the ECI frame i can be obtained based on the automatic identification of celestial bodies and the calculation of celestial apparent positions. Due to the influence of the measurement error, the transformation relationship is approximately described as follows.

(1) $${\tilde{R}}_{j}^{i}\approx C_{s}^{i}{\tilde{R}}_{j}^{s},(\;j=1,2,\cdots ,m)$$

where $C_{s}^{i}$ serves as the reference information for the initial alignment. According to the least squares theory, the optimal attitude matrix can be solved as follows.

(2) $$A=\sum_{j=1}^{m}\omega_{j}{\tilde{R}}_{j}^{i}{\left({\tilde{R}}_{j}^{s}\right)}^{T}$$

where $\omega_{j}$ is the weighting coefficient, in general, $\sum_{j=1}^{m}\omega_{j}=1$, in the case of equal-weighted average, $\omega_{j}=1/m$ or $\omega_{j}=1$. The singular value decomposition of matrix A can be expressed as $A=UDV^{T}$, then the optimal attitude matrix is described as follows.

(3) $$C_{s}^{i}=UV^{T}$$

## 4. Initial Alignment Model in the Navigation Frame

At present, initial alignment of the SINS is usually performed based on the static condition in the navigation frame. The velocity is taken as the measurement information, and initial alignment is performed based on the functional relationship between the velocity error and misalignment angle. The transfer alignment of the SINS is also usually performed in the navigation frame with the outputs of the Master Inertial Navigation System (MINS) as the reference information. However, the output error of the MINS will have a direct influence on the accuracy of the transfer alignment. In addition, the approximate error of the transfer alignment model due to the large dynamic deflection deformation angle $\gamma_{bba,bm}^{}$ will also degrade the alignment performance. The initial alignment model based on the attitude measurement of the CNS in the navigation frame is deduced as follows.

Under static or mooring conditions, the attitude error equation of the SINS can be simplified as

(4) $${\dot{\phi }}_{n,n^{\prime }}^{}=-(\omega_{in}^{n}\times ){\dot{\phi }}_{n,n^{\prime }}^{}-C_{bm}^{n^{\prime }}\varepsilon_{g}$$

where $\omega_{in}^{n}$ is the projection of the angular velocity in n from i to n, $\varepsilon_{g}^{}$ is the constant drift error of the gyroscope. According to the chain multiplication rules of the attitude transformation matrix, the following relations can be established.

(5) $$\begin{array}{ll} & C_{s}^{i}=C_{e}^{i}C_{n}^{e}C_{n^{\prime }}^{n}C_{bm}^{n^{\prime }}C_{ba}^{bm}C_{b}^{ba}C_{s}^{b}\\ \Rightarrow & {\left(C_{n}^{e}\right)}^{T}{\left(C_{e}^{i}\right)}^{T}C_{s}^{i}\\ & =(I+\phi_{n,n^{\prime }}\times )C_{bm}^{n^{\prime }}(I-\gamma_{ba,bm}^{}\times )(I-\mu_{b,ba}^{}\times )(I-\mu_{s,b}^{}\times ) \end{array}$$

Since both the installation error angle $\mu_{s,b}^{}$ and static deformation angle $\mu_{b,ba}^{}$ are constant values, they can be regarded as a whole for modeling estimation, expressed by $\mu_{s,ba}^{}$, where

(6) $$C_{b}^{ba}C_{s}^{b}=(I-\mu_{b,ba}^{}\times )(I-\mu_{s,b}^{}\times )=C_{s}^{ba}=(I-\mu_{s,ba}^{}\times )$$

Substituting Equation (6) into Equation (5) can be obtained as

(7) $$\begin{array}{cc} {\left(C_{n}^{e}\right)}^{T} & {\left(C_{e}^{i}\right)}^{T}C_{s}^{i}=(I+\phi_{n,n^{\prime }}\times )C_{bm}^{n^{\prime }}[I-(\gamma_{ba,bm}\times )-(\mu_{s,ba}^{}\times )]\\ & =C_{bm}^{n^{\prime }}+(\phi_{n,n^{\prime }}\times )C_{bm}^{n^{\prime }}-C_{bm}^{n^{\prime }}(\gamma_{ba,bm}^{}\times )-C_{bm}^{n^{\prime }}(\mu_{s,ba}^{}\times )\\ \Rightarrow & {\left(C_{n}^{e}\right)}^{T}{\left(C_{e}^{i}\right)}^{T}C_{s}^{i}{\left(C_{bm}^{n^{\prime }}\right)}^{T}\\ & =I+(\phi_{n,n^{\prime }}\times )-C_{bm}^{n^{\prime }}(\gamma_{ba,bm}^{}\times ){\left(C_{bm}^{n^{\prime }}\right)}^{T}-C_{bm}^{n^{\prime }}(\mu_{s,ba}^{}\times ){\left(C_{bm}^{n^{\prime }}\right)}^{T}\\ & \approx I+(\phi_{n,n^{\prime }}\times )-[(C_{bm}^{n^{\prime }}\gamma_{ba,bm}^{})\times ]-[(C_{bm}^{n^{\prime }}\mu_{s,ba}^{})\times ] \end{array}$$

The second-order term of error is omitted in the derivation, and the approximate treatment is as follows.

(8) $$[(C_{bm}^{n^{\prime }}\mu_{s,ba}^{})\times ]\approx C_{bm}^{n^{\prime }}(\mu_{s,ba}^{}\times ){\left(C_{bm}^{n^{\prime }}\right)}^{T}$$

That is, the projection of $\mu_{s,ba}^{}$ in frame ba is approximately the same as the projection in frame bm. Since the deflection between ba and bm is determined by the dynamic deflection angle $\gamma_{ba,bm}^{}$, the alignment accuracy will be affected to some extent when $\gamma_{ba,bm}^{}$ is larger. In Equation (7), $C_{s}^{i}$ is obtained from the CNS; $C_{e}^{i}$ is the time matrix, which can be obtained by accurate calculation under the guarantee of a high-precision time reference; $C_{bm}^{n^{\prime }}$ is the attitude matrix provided by the SINS in the calculated navigation frame $n^{\prime }$; $C_{n}^{e}$ is the position matrix, which is the known quantity under static conditions. Since it fails to obtain accurate position information under dynamic conditions, the model is only suitable for the static initial alignment.

The astronomical error angle is defined as ψ, which serves as the measurement information for the initial alignment, according to Equation (7), it can be expressed as

(9) $$\psi =\phi_{n,n^{\prime }}-C_{bm}^{n^{\prime }}\mu_{s,ba}^{}-C_{bm}^{n^{\prime }}\gamma_{ba,bm}^{}$$

## 5. Attitude Matching Initial Alignment Algorithm in the ECI Frame

The CNS can directly obtain the attitude matrix $C_{s}^{i}$ from frame i to frame s without the help of position information. Therefore, the one-step initial alignment of the SINS in full motion can be realized based on the attitude measurement provided by the CNS in the ECI frame.

### 5.1. Attitude Error Equation in the ECI Frame

$\omega_{i,bm}^{bm}$ is the theoretical angular velocity in frame bm, ${\tilde{\omega }}_{i,bm}^{bm}$ is the angular velocity measured by the gyroscope. So the measurement error of angular velocity can be described as

(10) $$\delta \omega_{i,bm}^{bm}={\tilde{\omega }}_{i,bm}^{bm}-\omega_{i,bm}^{bm}$$

The attitude matrix provided by the SINS in the ECI frame can be expressed as

(11) $$C_{bm}^{i^{\prime }}=C_{i}^{i^{\prime }}C_{bm}^{i}$$

To differentiate both sides of Equation (11) as

(12) $$\begin{array}{l} \;{\dot{C}}_{bm}^{i^{\prime }}={\dot{C}}_{i}^{i^{\prime }}C_{bm}^{i}+C_{i}^{i^{\prime }}{\dot{C}}_{bm}^{i}\\ \Rightarrow C_{bm}^{i^{\prime }}({\tilde{\omega }}_{i,bm}^{bm}\times )=(-{\dot{\phi }}_{i,i^{\prime }}^{}\times )C_{bm}^{i}+C_{i}^{i^{\prime }}C_{bm}^{i}(\omega_{i,bm}^{bm}\times )\\ \Rightarrow (-{\dot{\phi }}_{i,i^{\prime }}\times )C_{bm}^{i}=C_{bm}^{i^{\prime }}({\tilde{\omega }}_{i,bm}^{bm}\times )-C_{bm}^{i^{\prime }}(\omega_{i,bm}^{bm}\times )\\ \Rightarrow (-{\dot{\phi }}_{i,i^{\prime }}^{}\times )=C_{bm}^{i^{\prime }}[({\tilde{\omega }}_{i,bm}^{bm}-\omega_{i,bm}^{bm})\times ]{\left(C_{bm}^{i^{\prime }}\right)}^{T}C_{i}^{i^{\prime }}\\ =[(C_{bm}^{i^{\prime }}\delta \omega_{i,bm}^{bm})\times ](I-\phi_{i,i^{\prime }}^{}\times )\\ \approx [(C_{bm}^{i^{\prime }}\delta \omega_{i,bm}^{bm})\times ]\\ \Rightarrow {\dot{\phi }}_{i,i^{\prime }}^{}\approx -C_{bm}^{i^{\prime }}\delta \omega_{i,bm}^{bm} \end{array}$$

The second-order term of error is omitted in the derivation. With the attitude update, misalignment angle $\phi_{i,i\prime }$ will cause frame $i^{\prime }$ to gradually deviate from frame i, which can also be regarded as frame $bm^{\prime }$ to gradually deviate from frame bm. It can be expressed as

(13) $$\begin{array}{l} C_{bm}^{i^{\prime }}=C_{i}^{i^{\prime }}C_{bm}^{i}=C_{bm^{\prime }}^{i}=C_{bm}^{i}C_{bm^{\prime }}^{bm}\\ \Rightarrow (I-\phi_{i,i^{\prime }}^{}\times )C_{bm}^{i}=C_{bm}^{i}(I+\phi_{bm,bm^{\prime }}^{}\times )\\ \Rightarrow \;\phi_{bm,bm^{\prime }}^{}=-{\left(C_{bm}^{i}\right)}^{T}\phi_{i,i^{\prime }}^{} \end{array}$$

Similarly, the process that frame $bm^{\prime }$ gradually deviates from frame bm can also be regarded as frame $n^{\prime }$ gradually deviating from frame n. It can be expressed as

(14) $$\begin{array}{l} C_{bm}^{n^{\prime }}=C_{n}^{n^{\prime }}C_{bm}^{n}=C_{bm^{\prime }}^{n}=C_{bm}^{n}C_{bm^{\prime }}^{bm}\\ \Rightarrow (I-\phi_{n,n^{\prime }}^{}\times )C_{bm}^{n}=C_{bm}^{n}(I+\phi_{bm,bm^{\prime }}^{}\times )\\ \Rightarrow \;\phi_{n,n^{\prime }}^{}=-C_{bm}^{n}\phi_{bm,bm^{\prime }}^{}=C_{bm}^{n}{\left(C_{bm}^{i}\right)}^{T}\phi_{i,i^{\prime }}^{} \end{array}$$

### 5.2. Dynamic Deflection Deformation Model 

The hull dynamic deflection is caused by the interference of the external environment, which can be described by the second-order Markov process, and the three axial deformation processes of the SINS are assumed to be independent of each other. $\omega_{ba,bm}^{bm}$ is the angular velocity of deflection deformation. In the following formulas, each vector is represented by the shorthand form, omitting the upper and lower markers.

(15) $${\dot{\omega }}_{i}=-\beta_{i}^{2}\gamma_{i}-2\beta_{i}\omega_{i}+\eta_{i},\;(i=x,y,z)$$

where $\gamma ={\left[\gamma_{x}\;\gamma_{y}\;\gamma_{z}\right]}^{T}$ is the dynamic deflection angle, its mean square error is defined as $\sigma ={\left[\sigma_{x}\;\sigma_{y}\;\sigma_{z}\right]}^{T}$, and ${\dot{\gamma }}_{i}=\omega_{i}$. $\eta ={\left[\eta_{x}\;\eta_{y}\;\eta_{z}\right]}^{T}$ is white noise, its spectral density is $Q_{\eta }={\left[Q_{\eta_{x}}\;Q_{\eta_{y}}\;Q_{\eta_{z}}\right]}^{T}$; that is, $\eta ∼Ν(0,Q_{\eta })$. $\beta ={\left[\beta_{x}\;\beta_{y}\;\beta_{z}\right]}^{T}$ is the constant vector. The functional relation in $Q_{\eta }$, σ, and β can be described as $Q_{\eta_{i}}=4\beta_{i}^{3}\sigma_{i}^{2}$, where $\beta_{i}=2.146/\tau_{i}$, $\tau_{i}$ is the correlation time of each random process [34,35].

To define

(16) $$N_{\beta \gamma }^{}=\left[\begin{array}{l} -\beta_{x}^{2}\;0\;0\\ 0-\beta_{y}^{2}\;0\\ 0\;0\;-\beta_{z}^{2} \end{array}\right],N_{\beta \omega }^{}=\left[\begin{array}{l} -2\beta_{x}^{}\;0\;0\\ 0-2\beta_{y}^{}\;0\\ 0\;0\;-2\beta_{z}^{} \end{array}\right]$$

So Equation (15) can be converted to

(17) $$\dot{\omega }=N_{\beta \gamma }^{}\gamma +N_{\beta \omega }^{}\omega +\eta$$

### 5.3. Attitude Measurement Model

According to the chain multiplication rules of the attitude transformation matrix, the following relations can be established.

(18) $$\begin{array}{ll} C_{s}^{i} & =C_{i^{\prime }}^{i}C_{bm}^{i^{\prime }}C_{ba}^{bm}C_{s}^{ba}\\ & =(I+\phi_{i,i^{\prime }}^{}\times )C_{bm}^{i^{\prime }}(I-\gamma_{ba,bm}^{}\times )(I-\mu_{s,ba}^{}\times )\\ \Rightarrow & {\left(C_{bm}^{i^{\prime }}\right)}^{T}C_{s}^{i}\approx [I+[{\left(C_{bm}^{i^{\prime }}\right)}^{T}\phi_{i,i^{\prime }}^{}-\gamma_{ba,bm}^{}-\mu_{s,ba}^{}]\times ] \end{array}$$

The second-order term of the error is omitted in the derivation. $C_{s}^{i}$ is obtained from the CNS; $C_{bm}^{i^{\prime }}$ is the attitude matrix provided by the SINS in frame $i^{\prime }$. The astronomical error angle is defined as ψ, which serves as the measurement information for the initial alignment; according to equation (18), it can be expressed as

(19) $$\psi ={\left(C_{bm}^{i^{\prime }}\right)}^{T}\phi_{i,i\prime }-\gamma_{ba,bm}^{}-\mu_{s,ba}^{}$$

### 5.4. The Kalman Filter for the Attitude Matching Algorithm

#### 5.4.1. The State Equation

The 15-dimensional state vectors for the initial alignment in the ECI frame can be expressed as

(20) $$X={\left[\mu^{T}\;\gamma^{T}\;\omega^{T}\;\phi_{i,i^{\prime }}^{T}\;\varepsilon_{g}^{T}\right]}^{T}$$

The state equation in matrix form can be expressed as

(21) $$\dot{X}=FX+Gw$$

where

(22) $$F=\left[\begin{array}{l} 0_{3\times 3}\;\;0_{3\times 3}\;\;0_{3\times 3}\;\;0_{3\times 3}\;\;0_{3\times 3}\\ 0_{3\times 3}\;\;0_{3\times 3}\;\;I_{3\times 3}\;\;0_{3\times 3}\;\;0_{3\times 3}\;\;\\ 0_{3\times 3}\;\;N_{\beta \gamma }^{}\;\;N_{\beta \omega }^{}\;\;0_{3\times 3}\;\;0_{3\times 3}\\ 0_{3\times 3}\;\;0_{3\times 3}\;\;0_{3\times 3}\;\;0_{3\times 3}\;\;-C_{bm}^{i^{\prime }}\\ 0_{3\times 3}\;\;0_{3\times 3}\;\;0_{3\times 3}\;\;0_{3\times 3}\;\;0_{3\times 3} \end{array}\right]$$

(23) $$G=\left[\begin{array}{cc} 0_{3\times 3} & 0_{3\times 3}\\ 0_{3\times 3} & 0_{3\times 3}\\ I_{3\times 3} & 0_{3\times 3}\\ 0_{3\times 3} & -C_{bm}^{i^{\prime }}\\ 0_{3\times 3} & 0_{3\times 3} \end{array}\right],w=\left[\begin{array}{c} \eta \\ \omega_{g}^{} \end{array}\right]$$

$\omega_{g}^{}$ is the random walk error of the gyroscope.

#### 5.4.2. The Measurement Equation

According to Equation (19), the measurement equation can be converted to

(24) $$Z={\left(C_{bm}^{i^{\prime }}\right)}^{T}\phi_{i,i^{\prime }}^{}-\gamma_{ba,bm}^{}-\mu_{s,ba}^{}$$

The measurement equation in matrix form can be expressed as

(25) Z=HX+v

where v is the measurement noise vector of the CNS, H is the measurement matrix, which can be expressed as

(26) $$H=\left[-I_{3\times 3}-I_{3\times 3}\;\;0_{3\times 3}\;\;{\left(C_{bm}^{i^{\prime }}\right)}^{T}\;\;0_{3\times 3}\right]$$

where $I_{3\times 3}\;$ is the three-dimensional identity matrix. 

## 6. Attitude and Velocity Matching Algorithm in the ECI Frame

When the carrier is in static or mooring conditions; that is, the position of the carrier is known and there is no linear motion relative to the Earth, the velocity measurement information can be added to the measurement equation to further improve the observability of state vectors. The attitude error equation, attitude measurement equation, and dynamic deflection deformation model are the same as those in Section 5.

### 6.1. Velocity Error Equation in the ECI Frame

$f_{i,bm}^{bm}$ is the theoretical specific force in frame bm, ${\tilde{f}}_{i,bm}^{bm}$ is the specific force measured by the accelerometer. So the measurement error of the specific force can be described as

(27) $$\delta f_{i,bm}^{bm}={\tilde{f}}_{i,bm}^{bm}-f_{i,bm}^{bm}$$

The derivative of velocity with respect to time in the ECI frame can be expressed as

(28) $${\dot{\nu }}_{i,bm}^{i}=f_{i,bm}^{i}+G_{i,bm}^{i}$$

where $f_{i,bm}^{i}$ and $G_{i,bm}^{i}$ are the specific force and gravitation in the ECI frame, respectively, then the derivative of the velocity error with respect to time can be expressed as

(29) $$\delta {\dot{\nu }}_{i,bm}^{i}={\tilde{f}}_{i,bm}^{i}-f_{i,bm}^{i}+{\tilde{G}}_{i,bm}^{i}-G_{i,bm}^{i}$$

Although gravitation is mainly related to latitude and altitude, it has little variation near the surface of the Earth, so the gravitation error can be ignored for the ship or other near-surface carrier navigation. In addition, the specific force measured by the SINS is projected into frame bm, so Equation (29) can be converted to

(30) $$\begin{array}{ll} \delta {\dot{\nu }}_{i,bm}^{i} & =C_{bm}^{i^{\prime }}{\tilde{f}}_{i,bm}^{bm}-C_{bm}^{i}f_{i,bm}^{bm}\\ & =C_{bm}^{i^{\prime }}{\tilde{f}}_{i,bm}^{bm}-C_{i^{\prime }}^{i}C_{bm}^{i^{\prime }}f_{i,bm}^{bm}\\ & =C_{bm}^{i^{\prime }}{\tilde{f}}_{i,bm}^{bm}-(I+\phi_{i,i^{\prime }}^{}\times )C_{bm}^{i^{\prime }}({\tilde{f}}_{i,bm}^{bm}-\delta f_{i,bm}^{bm})\\ & =C_{bm}^{i^{\prime }}{\tilde{f}}_{i,bm}^{bm}-C_{bm}^{i^{\prime }}{\tilde{f}}_{i,bm}^{bm}+C_{bm}^{i^{\prime }}\delta f_{i,bm}^{bm}-(\phi_{i,i^{\prime }}^{}\times )C_{bm}^{i^{\prime }}{\tilde{f}}_{i,bm}^{bm}+(\phi_{i,i^{\prime }}^{}\times )C_{bm}^{i^{\prime }}\delta f_{i,bm}^{bm}\\ & =(C_{bm}^{i^{\prime }}{\tilde{f}}_{i,bm}^{bm}\times )\phi_{i,i^{\prime }}^{}+C_{bm}^{i^{\prime }}\delta f_{i,bm}^{bm} \end{array}$$

The second-order term of error $(\phi^{i,i\prime }\times )C_{bm}^{i^{\prime }}\delta f_{i,bm}^{bm}$ is omitted in derivation. It should be noted that when the carrier is stationary relative to the Earth, the velocity ${\bar{\nu }}_{ib}^{i}$ in the ECI frame is only the linear velocity caused by the Earth’s rotation, which can be expressed as

(31) $${\bar{\nu }}_{ib}^{i}=(\omega_{ie}^{i}\times )r_{ib}^{i}$$

$\omega_{ie}^{i}$ is the angular velocity of the Earth’s rotation and $r_{ib}^{i}$ is the Cartesian position in the ECI frame under static conditions, both of which are known.

### 6.2. The Kalman Filter for the Attitude and Velocity Matching Algorithm

#### 6.2.1. The State Equation

The 21-dimensional state vectors for the initial alignment in the ECI frame can be expressed as

(32) $$X={\left[\phi_{i,i^{\prime }}^{T}\;\delta v\;\varepsilon_{g}^{T}\nabla_{a}^{T}\;\mu^{T}\;\gamma^{T}\;\omega^{T}\right]}^{T}$$

where $\nabla_{a}^{}$ is the accelerometer bias. The state equation in matrix form can be expressed as

(33) $$\dot{X}=FX+Gw$$

where

(34) $$F=\left[\begin{array}{ccccccc} 0_{3\times 3} & 0_{3\times 3} & -C_{bm}^{i^{\prime }} & 0_{3\times 3} & 0_{3\times 3} & 0_{3\times 3} & 0_{3\times 3}\\ (C_{bm}^{i^{\prime }}{\tilde{f}}_{i,bm}^{bm})\times & 0_{3\times 3} & 0_{3\times 3} & C_{bm}^{i^{\prime }} & 0_{3\times 3} & 0_{3\times 3} & 0_{3\times 3}\\ 0_{3\times 3} & 0_{3\times 3} & 0_{3\times 3} & 0_{3\times 3} & 0_{3\times 3} & 0_{3\times 3} & 0_{3\times 3}\\ 0_{3\times 3} & 0_{3\times 3} & 0_{3\times 3} & 0_{3\times 3} & 0_{3\times 3} & 0_{3\times 3} & 0_{3\times 3}\\ 0_{3\times 3} & 0_{3\times 3} & 0_{3\times 3} & 0_{3\times 3} & 0_{3\times 3} & 0_{3\times 3} & 0_{3\times 3}\\ 0_{3\times 3} & 0_{3\times 3} & 0_{3\times 3} & 0_{3\times 3} & 0_{3\times 3} & 0_{3\times 3} & I_{3\times 3}\\ 0_{3\times 3} & 0_{3\times 3} & 0_{3\times 3} & 0_{3\times 3} & 0_{3\times 3} & N_{\beta \gamma }^{} & N_{\beta \omega }^{} \end{array}\right]$$

(35) $$G=\left[\begin{array}{ccc} -C_{bm}^{i^{\prime }} & 0_{3\times 3} & 0_{3\times 3}\\ 0_{3\times 3} & C_{bm}^{i^{\prime }} & 0_{3\times 3}\\ 0_{12\times 3} & 0_{12\times 3} & 0_{12\times 3}\\ 0_{3\times 3} & 0_{3\times 3} & I_{3\times 3} \end{array}\right],w=\left[\begin{array}{c} \omega_{g}^{}\\ \omega_{a}^{}\\ \eta \end{array}\right]$$

$\omega_{a}^{}$ is the random noise of the accelerometer.

#### 6.2.2. The Measurement Equation

The measurement equation for the attitude and velocity matching algorithm can be expressed as

(36) $$\begin{array}{l} Z_{\psi }={\left(C_{bm}^{i^{\prime }}\right)}^{T}\phi_{i,i^{\prime }}^{}-\gamma_{ba,bm}^{}-\mu_{s,ba}^{}\\ Z_{v}=\delta v \end{array}$$

The measurement equation in matrix form can be expressed as

(37) Z=HX+v

where v is the noise vector of the CNS and velocity measurement, H is measurement matrix, which can be expressed as

(38) $$H=\left[\begin{array}{ccccccc} {\left(C_{bm}^{i^{\prime }}\right)}^{T} & 0_{3\times 3} & 0_{3\times 3} & 0_{3\times 3} & -I_{3\times 3} & -I_{3\times 3} & 0_{3\times 3}\\ 0_{3\times 3} & I_{3\times 3} & 0_{3\times 3} & 0_{3\times 3} & 0_{3\times 3} & 0_{3\times 3} & 0_{3\times 3} \end{array}\right]$$

## 7. Experimental Verification and Discussion

### 7.1. The Simulation Experiment for the Initial Alignment

#### 7.1.1. The Simulation Experiment for the Initial Alignment in Quasi-Static Swing Base

In order to compare the performances of various initial alignment algorithms under static or mooring conditions, the simulation experiments in the quasi-static swing base are designed for the initial misalignment angles ${\left[0.2{}^{\circ}\;0.2{}^{\circ}\;2{}^{\circ}\right]}^{T}$ and ${\left[5{}^{\circ}5{}^{\circ}20{}^{\circ}\right]}^{T}$, respectively. The experimental conditions are as follows.

**Figure 2:**
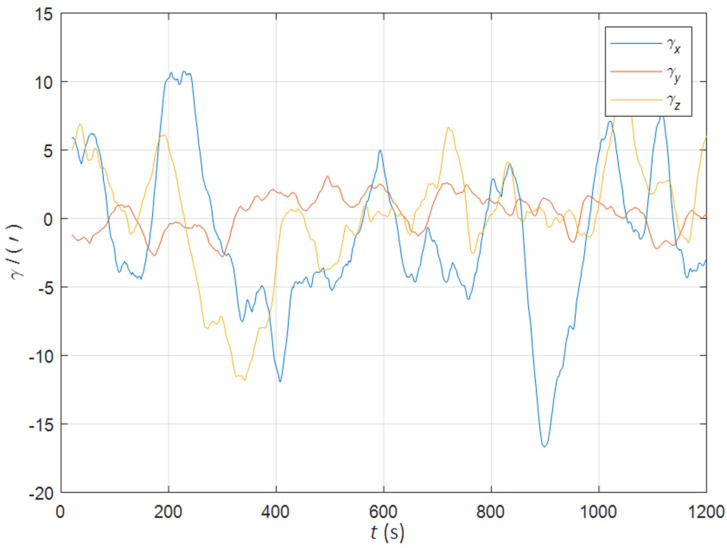
The curves of the dynamic deflection angle in the quasi-static swing base.

Initial position: φ 34°.00N, λ 108°.00E. Initial attitude: ${\left[0{}^{\circ}\;0{}^{\circ}\;0{}^{\circ}\right]}^{T}$. Swinging angular amplitudes (pitch, roll, yaw): ${\left[3{}^{\circ}\;2{}^{\circ}\;4{}^{\circ}\right]}^{T}$. Swinging angular periods: 12 s, 10 s, and 15 s. Inertial device performance of the SINS: gyro drift 0.01°/h, random walk $0.001{}^{\circ}/\sqrt{h}$, accelerometer bias 50 ug, random noise $5\;ug/\sqrt{s}$. The static deformation angle $\mu_{b,ba}^{}$: ${\left[30^{\prime }\;10^{\prime }\;20^{\prime }\right]}^{T}$. The mean square error of the dynamic deflection angle: ${\left[6^{\prime }\;2^{\prime }\;4^{\prime }\right]}^{T}$. The triaxial correlation times: 60 s, 60 s, 60 s. The curves of the dynamic deflection angle γ are shown as Figure 2.

**Figure 3:**
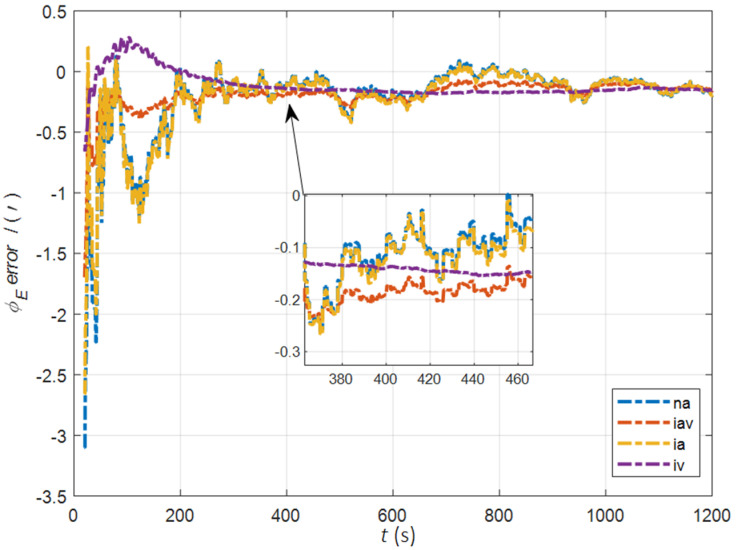
The error curves of $\phi_{E}$ in the cases of the initial misalignment angles ${\left[0.2{}^{\circ}\;0.2{}^{\circ}\;2{}^{\circ}\right]}^{T}$.

**Figure 4:**
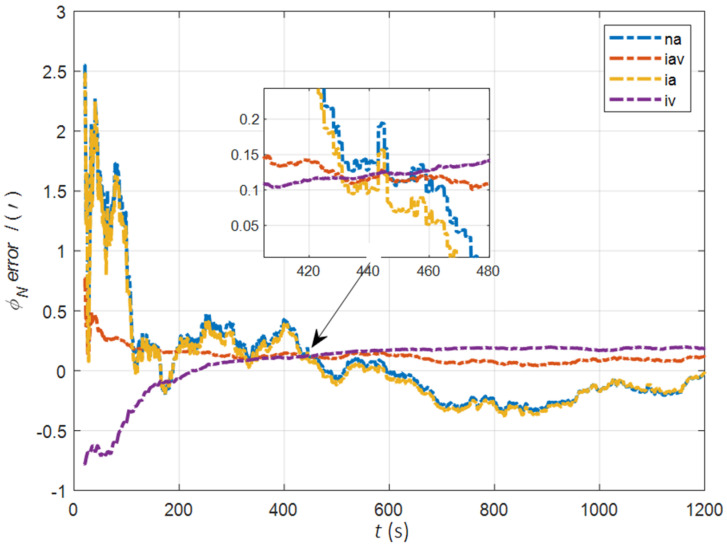
The error curves of $\phi_{N}$ in the cases of the initial misalignment angles ${\left[0.2{}^{\circ}\;0.2{}^{\circ}\;2{}^{\circ}\right]}^{T}$.

**Figure 5:**
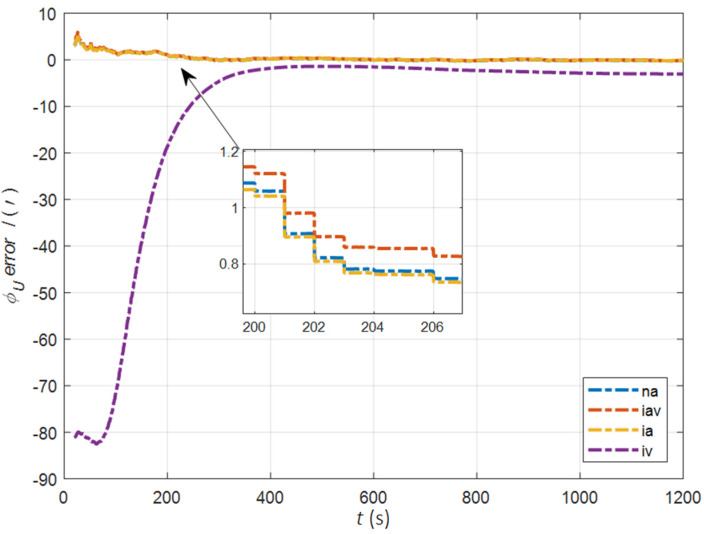
The error curves of $\phi_{U}$ in the cases of the initial misalignment angles ${\left[0.2{}^{\circ}\;0.2{}^{\circ}\;2{}^{\circ}\right]}^{T}$.

In the cases of the initial misalignment angles ${\left[0.2{}^{\circ}\;0.2{}^{\circ}\;2{}^{\circ}\right]}^{T}$, the error curves of the misalignment angles ϕ are shown in Figure 3, Figure 4 and Figure 5.

The data in Table 1 are the root mean square error (RMSE) values of ϕ from 200 to 1200 s for different initial alignment algorithms. In the cases of the initial misalignment angles ${\left[0.2{}^{\circ}\;0.2{}^{\circ}\;2{}^{\circ}\right]}^{T}$, the attitude and velocity matching algorithm in the ECI frame (iav) has the fastest alignment speed, the best stability, and the highest accuracy. Its initial alignment accuracy is better than 0.3′, in particular, the RMSE of the north alignment is 0.11′, which is obviously better than other algorithms. This is because the velocity measurement information further improves the observability of the misalignment angle, and effectively inhibits the influence of hull deformation on the accuracy of the initial alignment. The alignment speed and accuracy of the attitude matching algorithm in the navigation frame (na) and attitude matching algorithm in the ECI frame (ia) are similar. The horizontal alignment accuracy of the traditional velocity matching algorithm in the ECI frame (iv) is equivalent to that of other algorithms, but the RMSE of the azimuth misalignment angle reached 4.07′, which is obviously inferior to other algorithms.

**Figure 6:**
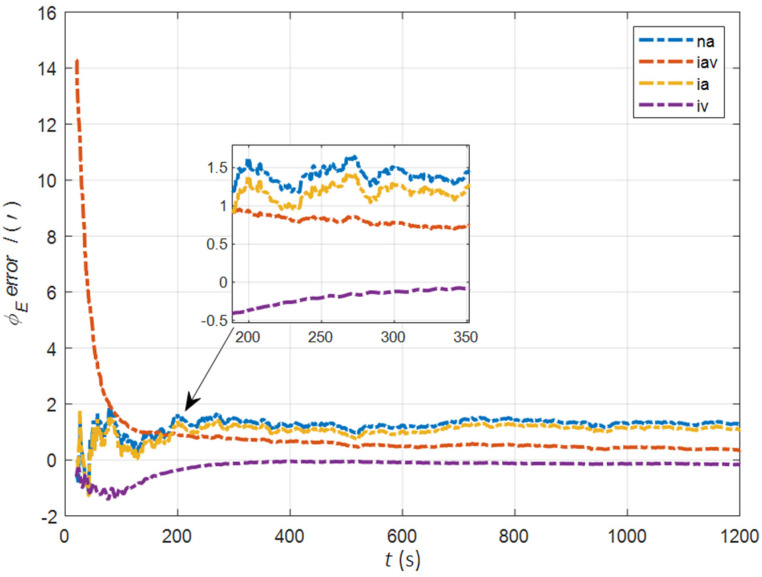
The error curves of $\phi_{E}$ in the cases of the initial misalignment angles ${\left[5{}^{\circ}5{}^{\circ}20{}^{\circ}\right]}^{T}$.

**Figure 7:**
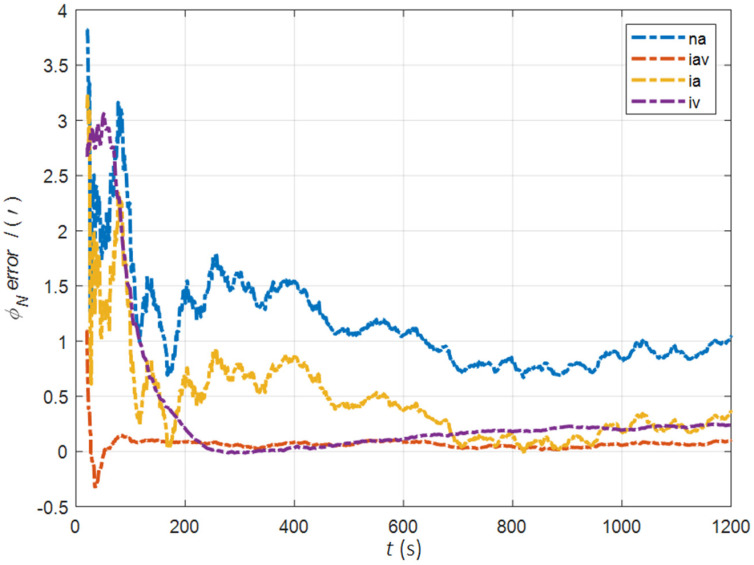
The error curves of $\phi_{N}$ in the cases of the initial misalignment angles ${\left[5{}^{\circ}5{}^{\circ}20{}^{\circ}\right]}^{T}$.

**Figure 8:**
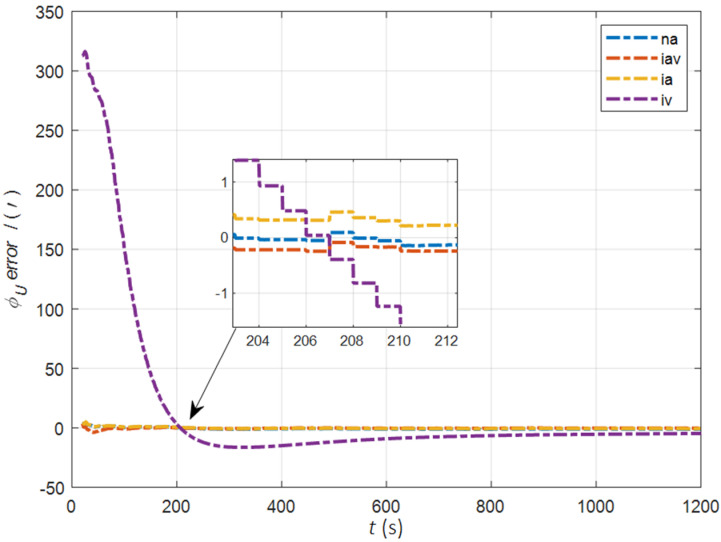
The error curves of $\phi_{U}$ in the cases of the initial misalignment angles ${\left[5{}^{\circ}5{}^{\circ}20{}^{\circ}\right]}^{T}$.

In the cases of the initial misalignment angles ${\left[5{}^{\circ}5{}^{\circ}20{}^{\circ}\right]}^{T}$, the error curves of the misalignment angles ϕ are shown in Figure 6, Figure 7 and Figure 8.

The data in Table 2 are the RMSE values of ϕ from 200 to 1200 s for different initial alignment algorithms. In the cases of the initial misalignment angles ${\left[5{}^{\circ}5{}^{\circ}20{}^{\circ}\right]}^{T}$, the traditional velocity matching algorithm in the ECI frame (iv) has better horizontal alignment speed and accuracy, but the azimuth alignment error is as high as 9.52′, which is obviously inferior to other algorithms. The alignment accuracy of attitude matching algorithm in the ECI frame (ia) is significantly better than that of attitude matching algorithm in the navigation frame (na). This is because ia avoids the influence of the approximate error caused by the dynamic deflection deformation angle, especially in the case of the large initial misalignment angle. For the attitude and velocity matching algorithm in the ECI frame (iav), the estimated accuracy of $\phi_{E}$ is better than 0.6′, the estimated accuracy of $\phi_{N}$ is better than 0.1′, and the estimated accuracy of $\phi_{U}$ is 0.3′. The alignment accuracy, speed and stability are the best among all algorithms, which are consistent with the experimental conclusion in the case of a relatively small initial misalignment angle.

#### 7.1.2. The Simulation Experiment for the Initial Alignment in the Dynamic Swing Base

**Figure 9:**
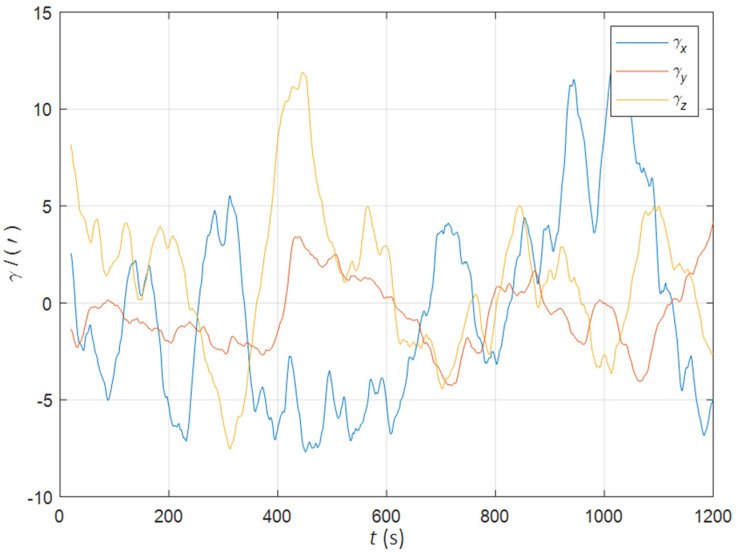
The curves of the dynamic deflection angle in the dynamic swing base.

The attitude matching algorithm in the ECI frame (ia) does not need the position as a priori information, so it can realize one-step alignment of the SINS in full motion state. In order to verify the performance of this algorithm, simulation experiments are designed for the initial misalignment angles ${\left[0.2{}^{\circ}\;0.2{}^{\circ}\;2{}^{\circ}\right]}^{T}$ and ${\left[5{}^{\circ}5{}^{\circ}20{}^{\circ}\right]}^{T}$ two cases respectively. The initial speed of uniform linear motion was set as 12 knots, and other experimental conditions were the same as in Section 7.1. The dynamic deflection angle curves are shown as Figure 9.

**Figure 10:**
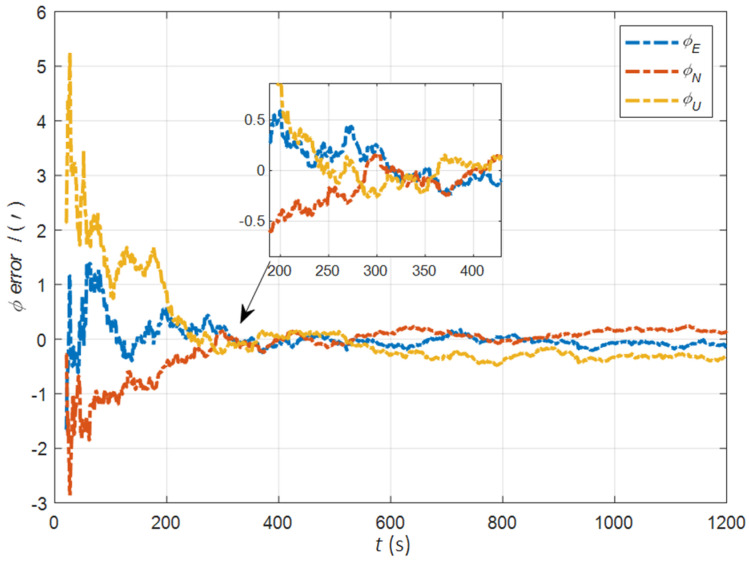
The error curves of ϕ in the cases of the initial misalignment angles ${\left[0.2{}^{\circ}\;0.2{}^{\circ}\;2{}^{\circ}\right]}^{T}$.

**Figure 11:**
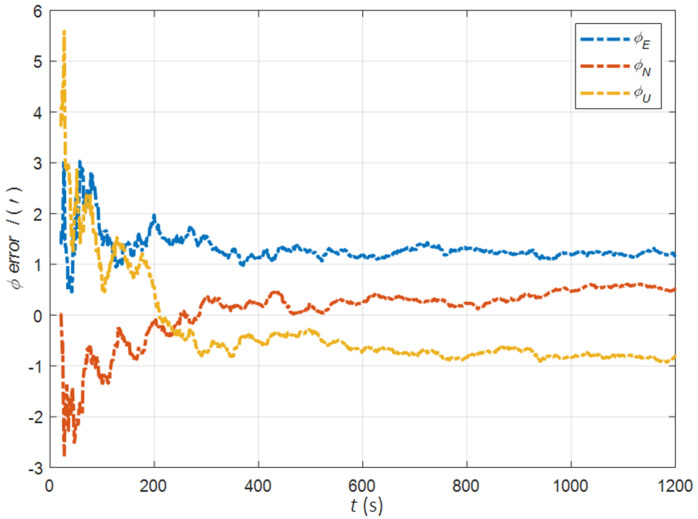
The error curves of ϕ in the cases of the initial misalignment angles ${\left[5{}^{\circ}5{}^{\circ}20{}^{\circ}\right]}^{T}$.

In the case of large and small initial misalignment angles, the error curves of ϕ are shown in Figure 10 and Figure 11.

The data in Table 3 are the RMSE values of ϕ in 200–1200 s under two initial misalignment angles. As can be seen from the above figures and tables, the estimated errors of the triaxial misalignment angle have converged rapidly since 200 s. In the cases of the initial misalignment angles ${\left[0.2{}^{\circ}\;0.2{}^{\circ}\;2{}^{\circ}\right]}^{T}$, the estimation accuracy of the horizontal misalignment angle is better than 0.2′, and that of the azimuth misalignment angle is better than 0.3′. In the cases of the initial misalignment angles ${\left[5{}^{\circ}5{}^{\circ}20{}^{\circ}\right]}^{T}$, the estimated accuracy of $\phi_{E}$ is better than 1.3′, the estimated accuracy of $\phi_{N}$ is better than 0.4′, and the estimated accuracy of $\phi_{U}$ is better than 0.7′.

### 7.2. The Simulation Experiment for Deformation Measurement

#### 7.2.1. The Simulation Experiment for Deformation Measurement in Quasi-Static Swing Base

**Figure 12:**
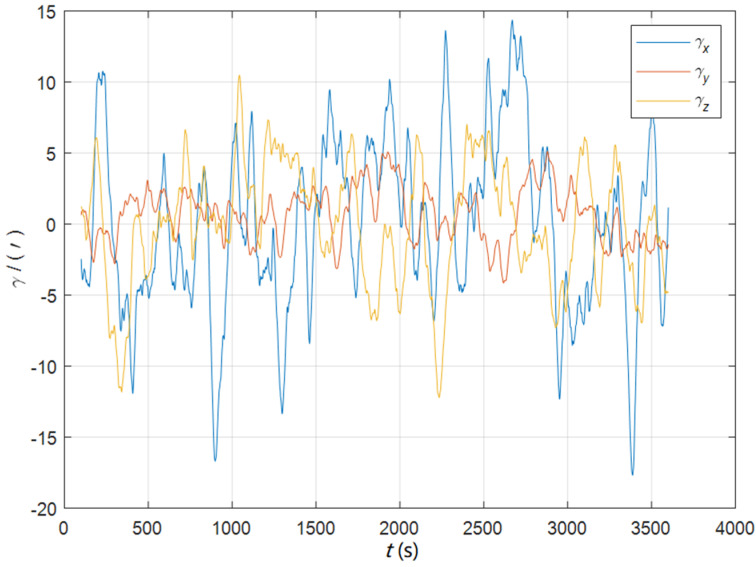
The curves of the dynamic deflection angle in the quasi-static swing base.

The algorithm proposed in this paper can realize the hull deformation measurement at the same time of the initial alignment, so in order to verify the performance of attitude matching and the attitude velocity matching algorithm for deformation measurements under static or mooring conditions, the simulation experiments in a quasi-static swing base were designed for the initial misalignment angles ${\left[0.5^{\prime }\;0.5^{\prime }\;10^{\prime }\right]}^{T}$ and ${\left[5{}^{\circ}5{}^{\circ}20{}^{\circ}\right]}^{T}$, respectively, in 3600 s. Other experimental conditions were the same as in Section 7.1. The dynamic deflection angle curves are shown as Figure 12.

**Figure 13:**
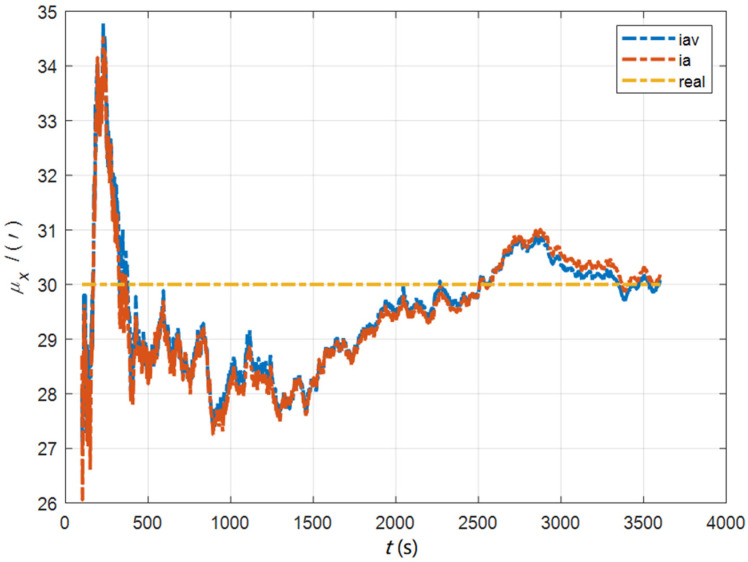
The curves of $\mu_{x}$ in the cases of the initial misalignment angles ${\left[0.5^{\prime }\;0.5^{\prime }\;10^{\prime }\right]}^{T}$.

**Figure 14:**
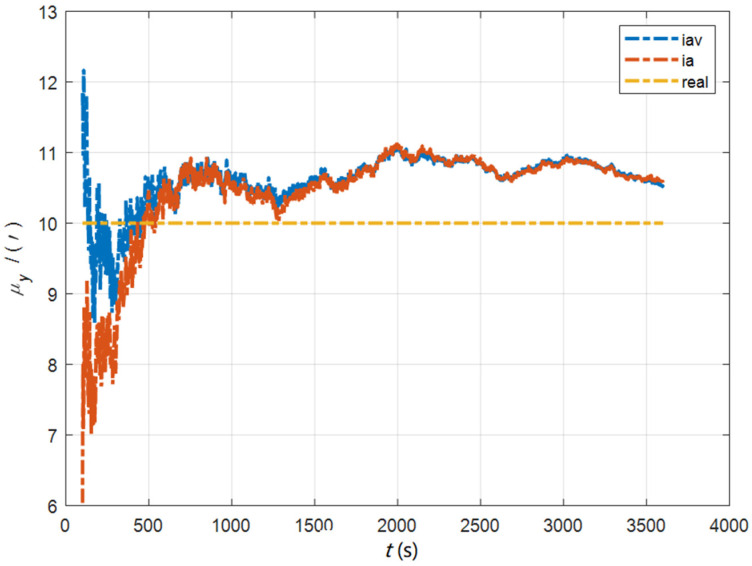
The curves of $\mu_{y}$ in the cases of the initial misalignment angles ${\left[0.5^{\prime }\;0.5^{\prime }\;10^{\prime }\right]}^{T}$.

**Figure 15:**
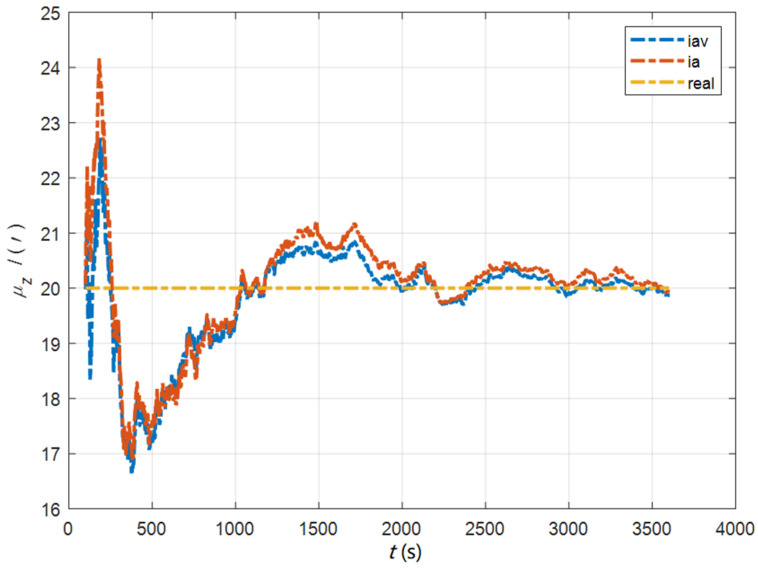
The curves of $\mu_{z}$ in the cases of the initial misalignment angles ${\left[0.5^{\prime }\;0.5^{\prime }\;10^{\prime }\right]}^{T}$.

In the cases of the initial misalignment angles ${\left[0.5^{\prime }\;0.5^{\prime }\;10^{\prime }\right]}^{T}$, the curves of the static deformation angle μ are shown in Figure 13, Figure 14 and Figure 15.

**Figure 16:**
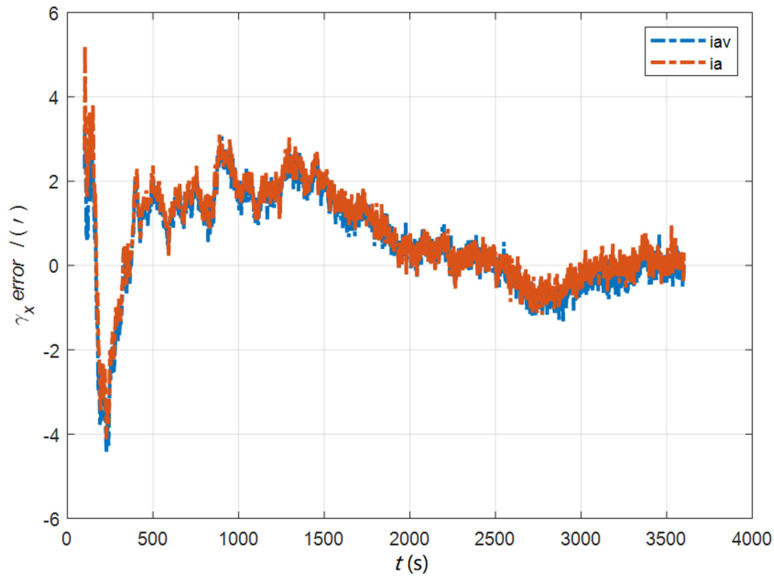
The error curves of $\gamma_{x}$ in the cases of the initial misalignment angles ${\left[0.5^{\prime }\;0.5^{\prime }\;10^{\prime }\right]}^{T}$.

**Figure 17:**
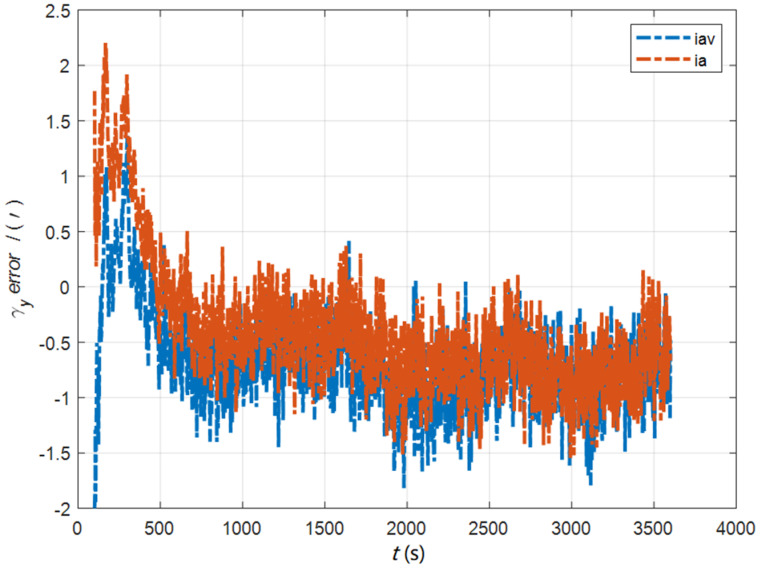
The error curves of $\gamma_{y}$ in the cases of the initial misalignment angles ${\left[0.5^{\prime }\;0.5^{\prime }\;10^{\prime }\right]}^{T}$.

**Figure 18:**
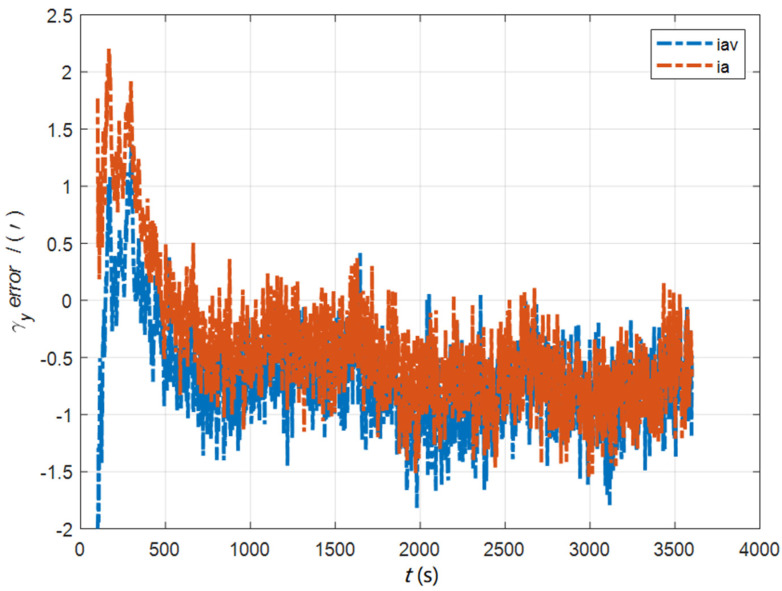
The error curves of $\gamma_{z}$ in the cases of the initial misalignment angles ${\left[0.5^{\prime }\;0.5^{\prime }\;10^{\prime }\right]}^{T}$.

In the cases of the initial misalignment angles ${\left[0.5^{\prime }\;0.5^{\prime }\;10^{\prime }\right]}^{T}$, the error curves of the dynamic deflection deformation angle γ are shown in Figure 16, Figure 17 and Figure 18.

The data in Table 4 are the RMSE values of μ and γ from 2800 to 3600 s for the attitude and velocity matching algorithm (iav) and the attitude matching algorithm (ia). It can be seen from the charts above, in the case of the small misalignment angle, the deformation measurement speed and accuracy of the two algorithms are relatively close. For the measurement of the static deformation angle μ, iav has better accuracy than ia in $\mu_{x}$ and $\mu_{z}$, which is about 6“, and the accuracies of the two algorithms in $\mu_{y}$ are similar. For the measurement of the dynamic deflection angle γ, the accuracies of the two algorithms are similar, and the difference is no more than 3“.

Based on the analysis of the above, since the velocity measurement information further improves the observability of the misalignment angle, and effectively inhibits the influence of the estimation error of the misalignment angle on the accuracy of the deformation measurement, algorithm iav has better deformation measurement performance; within one hour, the measurement accuracy of $\mu_{x}$ and $\mu_{z}$ can converge to less than 0.4′, and that of $\mu_{y}$ can converge to less than 0.8′. The measurement accuracy of $\gamma_{x}$ and $\gamma_{z}$ can converge to less than 0.5′, and the measurement accuracy of $\gamma_{y}$ can converge to less than 0.9′.

**Figure 19:**
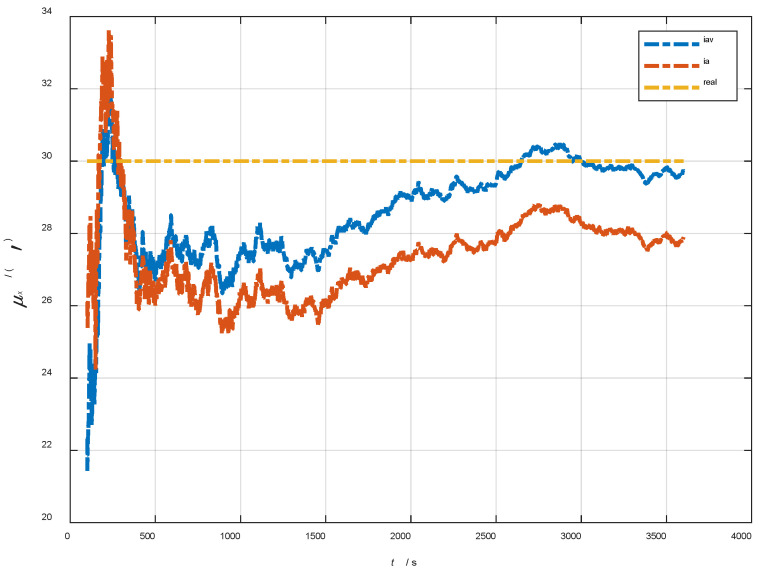
The curves of $\mu_{x}$ in the cases of the initial misalignment angles ${\left[5{}^{\circ}5{}^{\circ}20{}^{\circ}\right]}^{T}$.

**Figure 20:**
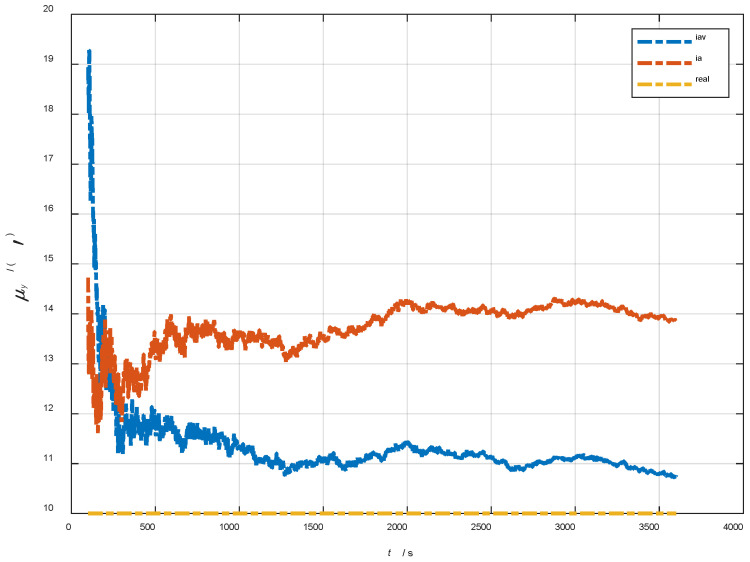
The curves of $\mu_{y}$ in the cases of the initial misalignment angles ${\left[5{}^{\circ}5{}^{\circ}20{}^{\circ}\right]}^{T}$.

**Figure 21:**
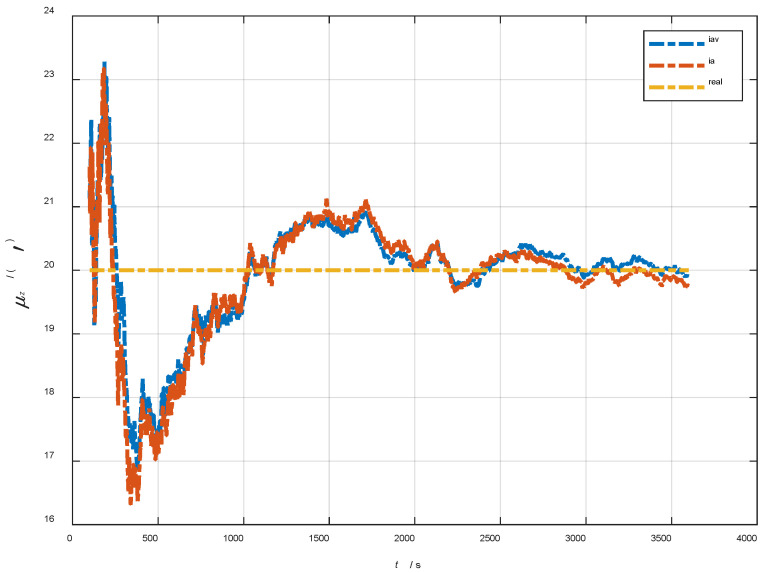
The curves of $\mu_{z}$ in the cases of the initial misalignment angles ${\left[5{}^{\circ}5{}^{\circ}20{}^{\circ}\right]}^{T}$.

In the cases of the initial misalignment angles ${\left[5{}^{\circ}5{}^{\circ}20{}^{\circ}\right]}^{T}$, the curves of the static deformation angle μ are shown in Figure 19, Figure 20 and Figure 21.

**Figure 22:**
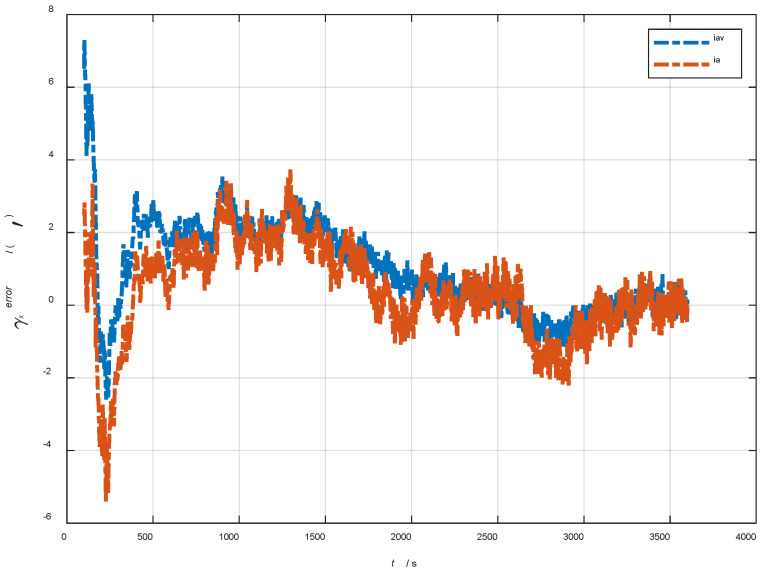
The error curves of $\gamma_{x}$ in the cases of the initial misalignment angles ${\left[5{}^{\circ}5{}^{\circ}20{}^{\circ}\right]}^{T}$.

**Figure 23:**
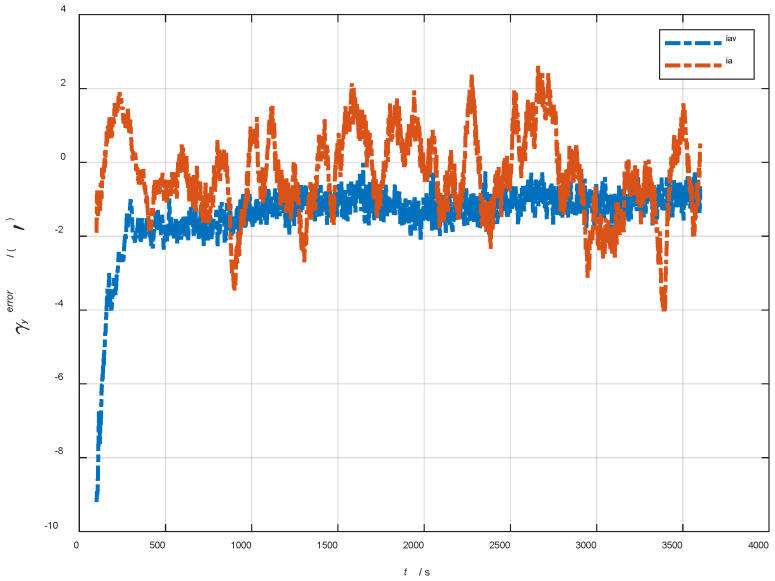
The error curves of $\gamma_{y}$ in the cases of the initial misalignment angles ${\left[5{}^{\circ}5{}^{\circ}20{}^{\circ}\right]}^{T}$.

**Figure 24:**
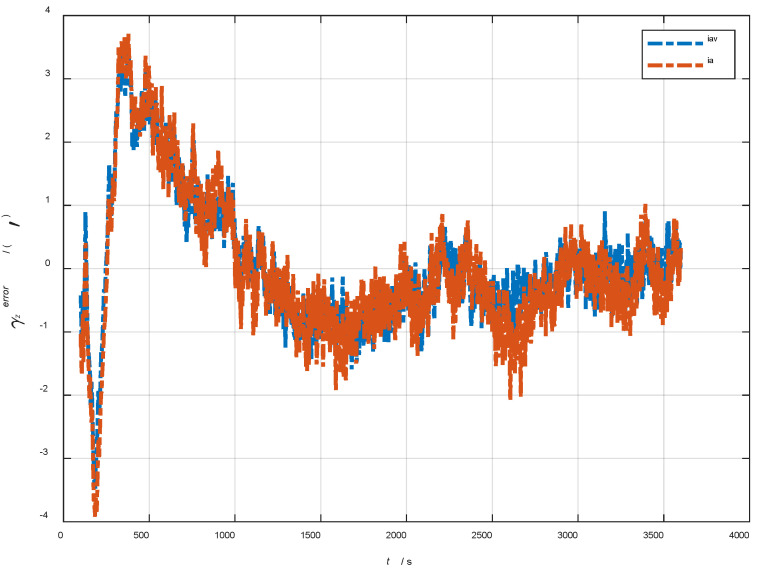
The error curves of $\gamma_{z}$ in the cases of the initial misalignment angles ${\left[5{}^{\circ}5{}^{\circ}20{}^{\circ}\right]}^{T}$.

In the cases of the initial misalignment angles ${\left[5{}^{\circ}5{}^{\circ}20{}^{\circ}\right]}^{T}$, the error curves of the dynamic deflection deformation angle γ are shown in Figure 22, Figure 23 and Figure 24.

The data in Table 5 are the RMSE values of μ and γ from 2800 to 3600 s for the attitude and velocity matching algorithm (iav) and attitude matching algorithm (ia). It can be seen from the charts above that, in the case of the large misalignment angle, iav has a higher deformation measurement accuracy, and its reasons and conclusions are consistent with the case of the small misalignment angle. Within one hour, the measurement accuracy of $\mu_{x}$ and $\mu_{z}$ can converge to less than 0.3′, and that of $\mu_{y}$ can converge to less than 1.0′. The measurement accuracy of $\gamma_{x}$ and $\gamma_{z}$ can converge to less than 0.4′, and that of $\gamma_{y}$ can converge to less than 1.0′.

#### 7.2.2. The Simulation Experiment for Deformation Measurement in the Dynamic Swing Base

**Figure 25:**
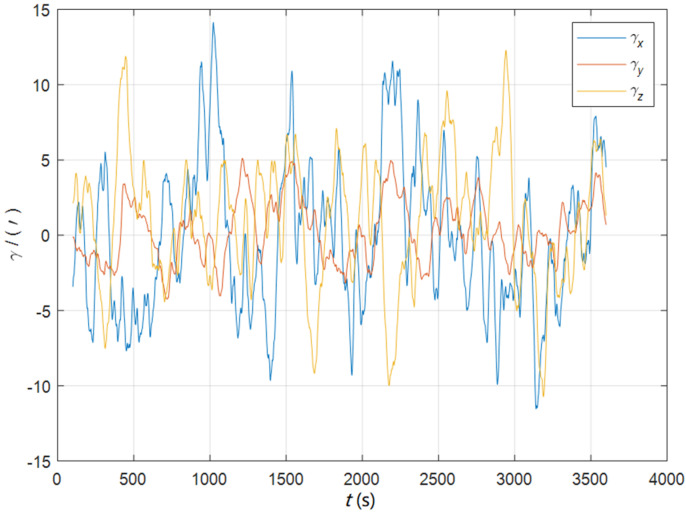
The curves of the dynamic deflection angle in the dynamic swing base.

The attitude matching algorithm in the ECI frame (ia) does not need the position as a priori information, so it can also realize hull deformation measurements in a full motion state. In order to verify the performance of this algorithm, simulation experiments were designed for the initial misalignment angles ${\left[0.5^{\prime }\;0.5^{\prime }\;10^{\prime }\right]}^{T}$ and ${\left[5{}^{\circ}5{}^{\circ}20{}^{\circ}\right]}^{T}$, two cases, respectively, in 3600 s. Other experimental conditions were the same as in Section 7.1. The dynamic deflection angle curves are shown as Figure 25.

**Figure 26:**
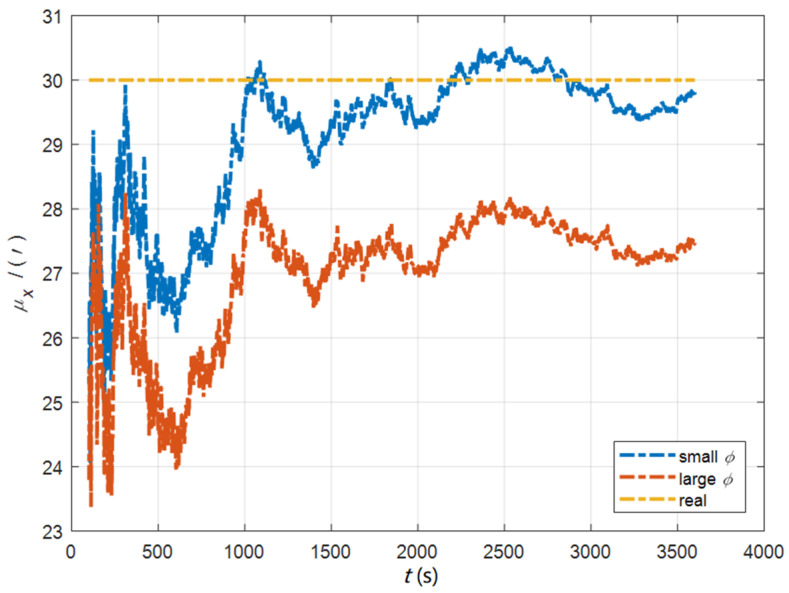
The curves of $\mu_{x}$ in the case of large and small initial misalignment angles.

**Figure 27:**
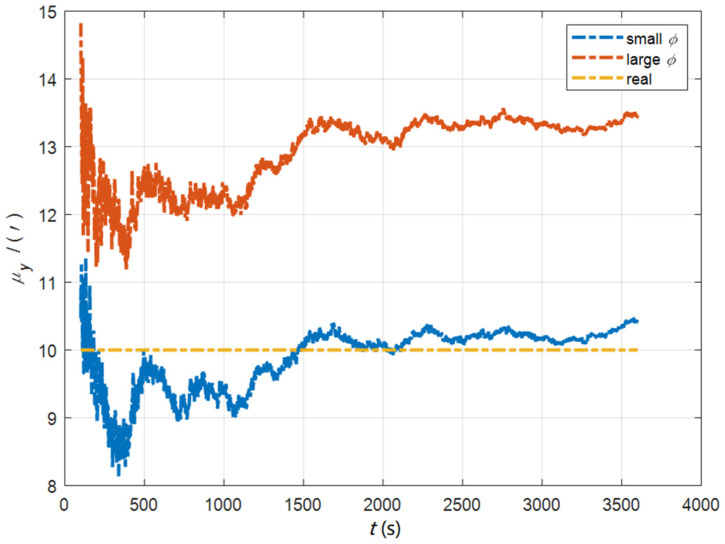
The curves of $\mu_{y}$ in the case of large and small initial misalignment angles.

**Figure 28:**
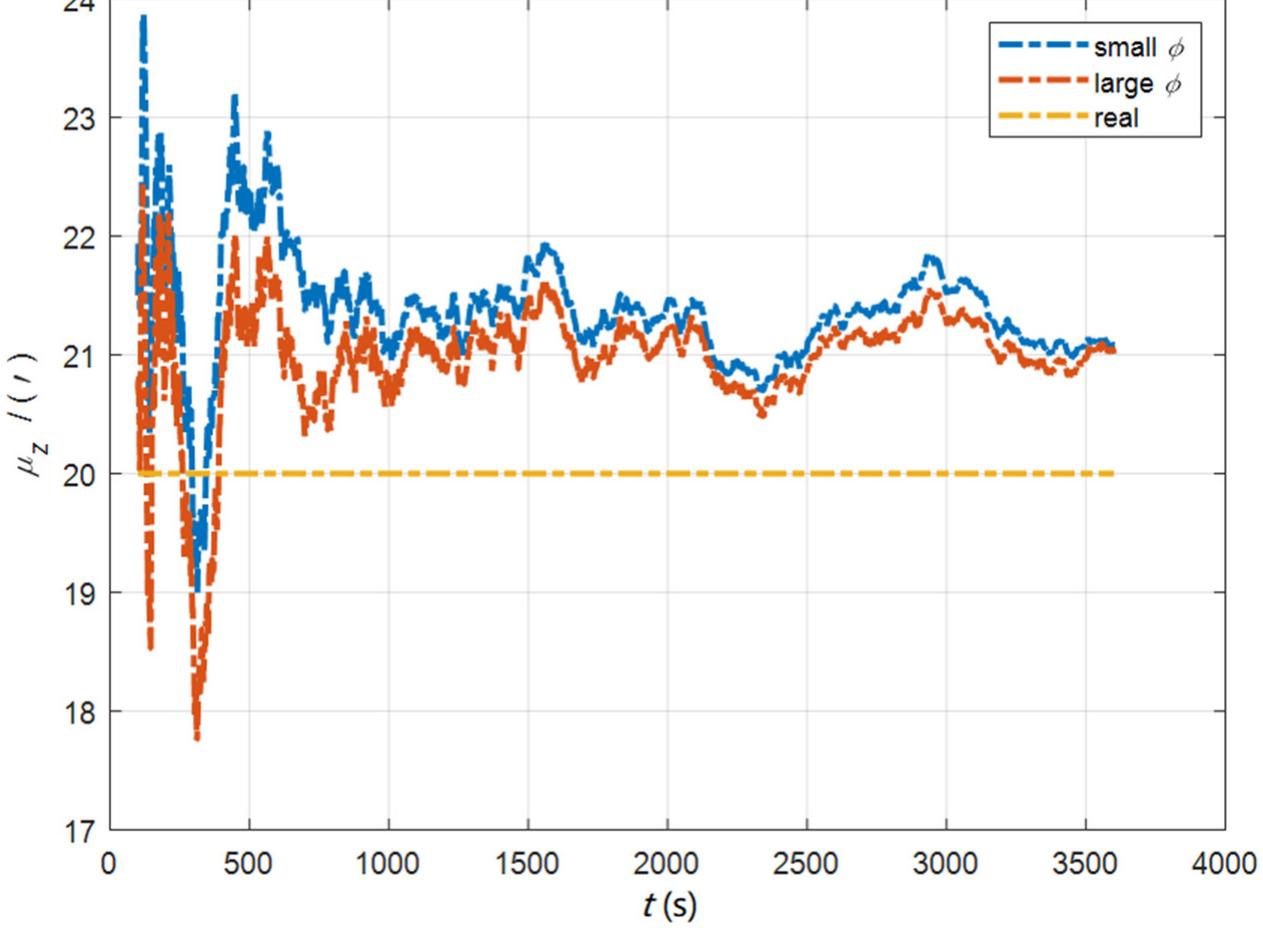
The curves of $\mu_{z}$ in the case of large and small initial misalignment angles.

In the case of large and small initial misalignment angles, the curves of the static deformation angle μ are shown in Figure 26, Figure 27 and Figure 28.

**Figure 29:**
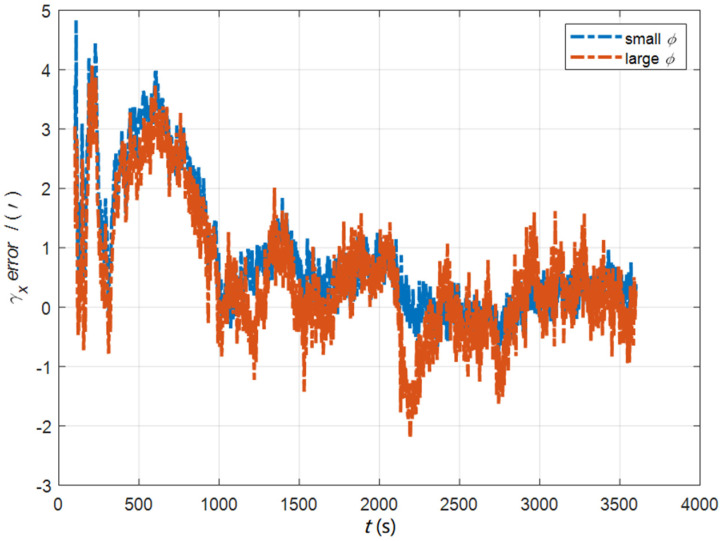
The error curves of $\gamma_{x}$ in the case of large and small initial misalignment angles.

**Figure 30:**
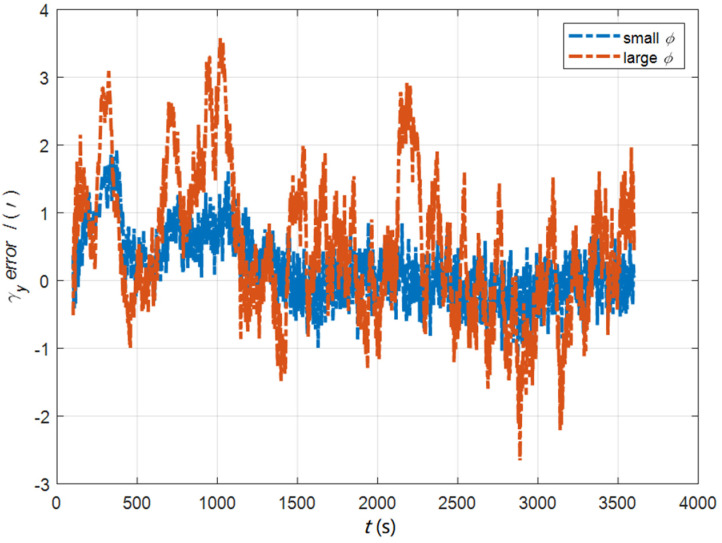
The error curves of $\gamma_{y}$ in the case of large and small initial misalignment angles.

**Figure 31:**
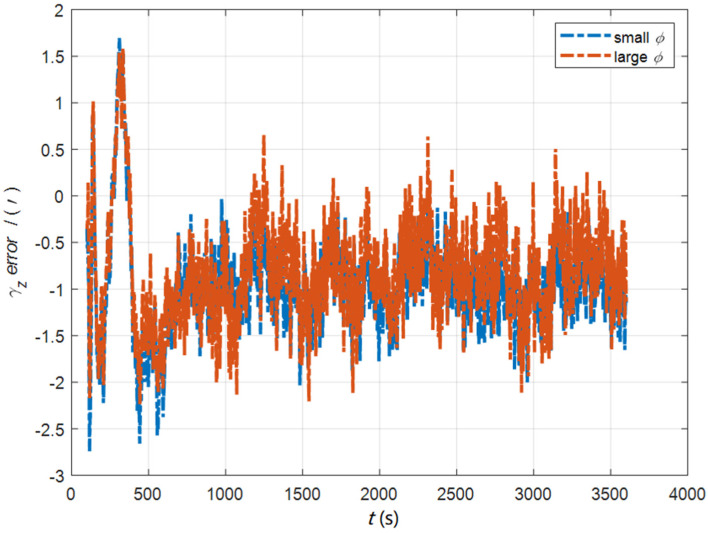
The error curves of $\gamma_{z}$ in the case of large and small initial misalignment angles.

In the case of large and small initial misalignment angles, the error curves of the dynamic deflection deformation angle γ are shown in Figure 29, Figure 30 and Figure 31.

The data in Table 6 are the RMSE values of μ and γ from 2800 to 3600 s for the attitude matching algorithm (ia) in the dynamic swing base. It can be seen from the charts above that, in the case of the small misalignment angle, ia has better measurement accuracy and speed in $\mu_{x}$, $\mu_{y}$, $\gamma_{x}$, and $\gamma_{y}$, which has significant advantages compared with the case of the large misalignment angle. However, the measurement accuracy and speed in $\mu_{z}$ and $\gamma_{z}$ are inferior to that of the large misalignment angle. Based on the analysis of the above, under the dynamic conditions, ia has better deformation measurement performance in the case of the small misalignment angle. Within one hour, the measurement accuracy of $\mu_{x}$ and $\mu_{y}$ can converge to less than 0.4′, and that of $\mu_{z}$ can converge to less than 1.4′. The measurement accuracy of $\gamma_{x}$ and $\gamma_{y}$ can converge to less than 0.4′, and the measurement accuracy of $\gamma_{z}$ can converge to less than 1.1′.

## 8. Conclusions

In this paper, the initial alignment algorithm in the ECI frame based on attitude measurement of the CNS is proposed, which can realize a fast and high-precision initial alignment of the SINS installed in various parts of the carrier under both dynamic and static conditions, and the algorithm can also realize the hull deformation measurement at the same time of the initial alignment. Since the high-precision astrometric attitude is directly used as the reference information for the initial alignment, compared with the traditional initial alignment algorithm, the algorithms proposed in this paper have better performances in alignment accuracy, speed, and stability. The experimental results show that under the condition of a quasi-static swing base, the attitude and velocity matching algorithm in the ECI frame has good estimation stability, and the initial alignment accuracy is better than 0.6′. Under the dynamic conditions, the attitude matching algorithm in the ECI frame can achieve alignment accuracy better than 1.3′, and the algorithm is not restricted by the carrier motion state. Under the condition of the quasi-static swing base, the alignment accuracy of this algorithm is still better than 1.2′, which can meet the rapid and high-precision initial alignment requirements of the SINS. In addition, the CNS is immune to electromagnetic interference and has no navigation error accumulation over time, such as the SINS, so the algorithms based on the CNS have important military application value and wider applicability.

## Figures and Tables

**Table 1:** RMSE values of $\phi_{}$ in the cases of the initial misalignment angles ${\left[0.2{}^{\circ}\;0.2{}^{\circ}\;2{}^{\circ}\right]}^{T}$.

Method	$\phi_{E}(\text{′})$	$\phi_{N}(\text{′})$	$\phi_{U}(\text{′})$
na	0.15	0.22	0.21
iav	0.16	0.11	0.23
ia	0.16	0.22	0.21
iv	0.15	0.16	4.07

**Table 2:** RMSE values of $\phi_{}$ in the cases of the initial misalignment angles ${\left[5{}^{\circ}5{}^{\circ}20{}^{\circ}\right]}^{T}$.

Method	$\phi_{E}(\text{′})$	$\phi_{N}(\text{′})$	$\phi_{U}(\text{′})$
na	1.32	1.08	0.85
iav	0.56	0.07	0.30
ia	1.12	0.43	0.49
iv	0.13	0.16	9.52

**Table 3:** RMSE values of ϕ in the cases of large and small initial misalignment angles.

Initial ϕ	$\phi_{E}(\text{′})$	$\phi_{N}(\text{′})$	$\phi_{U}(\text{′})$
${\left[0.2{}^{\circ}\;0.2{}^{\circ}\;2{}^{\circ}\right]}^{T}$	0.12	0.15	0.27
${\left[5{}^{\circ}\;5{}^{\circ}\;20{}^{\circ}\right]}^{T}$	1.26	0.35	0.68

**Table 4:** RMSE values of μ and γ in the cases of the initial misalignment angles ${\left[0.5^{\prime }\;0.5^{\prime }\;10^{\prime }\right]}^{T}$.

**Method**	$\mu_{x}(\text{′})$	$\mu_{y}\;(\text{′})$	$\mu_{z}\;(\text{′})$	$\gamma_{x}(\text{′})$	$\gamma_{y}\;(\text{′})$	$\gamma_{z}\;(\text{′})$
iav	0.36	0.73	0.10	0.43	0.85	0.29
ia	0.48	0.71	0.20	0.39	0.81	0.26

**Table 5:** RMSE values of μ and γ in the cases of the initial misalignment angles ${\left[5{}^{\circ}5{}^{\circ}20{}^{\circ}\right]}^{T}$.

**Method**	$\mu_{x}(\text{′})$	$\mu_{y}\;(\text{′})$	$\mu_{z}\;(\text{′})$	$\gamma_{x}(\text{′})$	$\gamma_{y}\;(\text{′})$	$\gamma_{z}\;(\text{′})$
iav	0.29	0.98	0.11	0.36	1.00	0.27
ia	1.92	4.09	0.13	0.76	1.47	0.39

**Table 6:** RMSE values of μ and γ in the case of the large and small initial misalignment angles.

Initial ϕ	$\mu_{x}(\text{′})$	$\mu_{y}\;(\text{′})$	$\mu_{z}\;(\text{′})$	$\gamma_{x}(\text{′})$	$\gamma_{y}\;(\text{′})$	$\gamma_{z}\;(\text{′})$
${\left[0.5^{\prime }\;0.5^{\prime }\;10^{\prime }\right]}^{T}$	0.36	0.24	1.35	0.32	0.29	1.05
${\left[5{}^{\circ}\;5{}^{\circ}\;20{}^{\circ}\right]}^{T}$	2.59	3.33	1.14	0.47	0.85	0.91
